# Exploring the Preventive Potential of Vitamin D against Respiratory Infections in Preschool-Age Children: A Cross-Sectional Study

**DOI:** 10.3390/nu16111595

**Published:** 2024-05-23

**Authors:** Oana Silvana Sarau, Hari Charan Rachabattuni, Sai Teja Gadde, Sai Praveen Daruvuri, Larisa Mihaela Marusca, Florin George Horhat, Ariadna Petronela Fildan, Elena Tanase, Catalin Prodan-Barbulescu, Delia Ioana Horhat

**Affiliations:** 1 Department V, Internal Medicine, Discipline of Hematology, “Victor Babes” University of Medicine and Pharmacy Timisoara, Eftimie Murgu Square 2, 300041 Timisoara, Romania; oana.sarau@umft.ro; 2 Doctoral School, ‘’Victor Babes’’ University of Medicine and Pharmacy Timisoara, Eftimie Murgu Square 2, 300041 Timisoara, Romania; tanase.elena@umft.ro (E.T.); catalin.prodan-barbulescu@umft.ro (C.P.-B.); 3 Faculty of General Medicine, Dr. Y.S.R. University of Health Sciences, Vijayawada 520008, India; haricharanrachabattuni@gmail.com; 4 Faculty of General Medicine, All India Institute of Medical Sciences (AIIMS), Mangalagiri 522503, India; gaddesai0626@gmail.com; 5 Faculty of General Medicine, Bukovinian State Medical University, Teatralna Square, 2, 58002 Chernivtsi, Ukraine; praveenzzz39@gmail.com; 6 Laboratory Medicine, “Louis Turcanu” Emergency Hospital for Children, Doctor Iosif Nemoianu Street, 300011 Timisoara, Romania; 7 Department of Microbiology, Multidisciplinary Research Center on Antimicrobial Resistance (MULTI-REZ), “Victor Babes” University of Medicine and Pharmacy, 300041 Timisoara, Romania; horhat.florin@umft.ro; 8 Department of Pulmonology, Faculty of Medicine, “Ovidius” University of Constanta, 900470 Constanta, Romania; petronela.fildan@365.univ-ovidius.ro; 9 Department of Anatomy and Embryology, Discipline of Pulmonology, “Victor Babes” University of Medicine and Pharmacy Timisoara, Eftimie Murgu Square 2, 300041 Timisoara, Romania; 10 IInd Surgery Clinic, “Victor Babes” University of Medicine and Pharmacy Timisoara, Eftimie Murgu Square 2, 300041 Timisoara, Romania; 11 Department of Ear-Nose-Throat, “Victor Babes” University of Medicine and Pharmacy, 300041 Timisoara, Romania; horhat.ioana@umft.ro

**Keywords:** vitamin D, children, blood tests, respiratory infections

## Abstract

Recent studies hypothesized that vitamin D supplementation and subsequent higher 25(OH)D serum levels could protect against respiratory infections in children. This cross-sectional study, conducted from May 2022 to December 2023 in Timisoara, Romania, aimed to evaluate the potential influence of vitamin D supplementation on the incidence of respiratory infections among preschool-age children. This study examined 215 children over 18 months who were split into a group of patients with recurrent respiratory infections (*n* = 141) and another group of patients with only one respiratory tract infection in the past 12 months (*n* = 74). Patients were evaluated based on their serum vitamin D levels 25(OH)D, demographic characteristics, and health outcomes. The study identified that preschool-age children with recurrent infections had significantly lower mean vitamin D concentrations (24.5 ng/mL) compared to the control group (29.7 ng/mL, *p* < 0.001). Additionally, a higher proportion of vitamin D deficiency was observed among children with recurrent infections in the past 12 months. Notably, vitamin D supplementation above 600 IU/week significantly reduced the likelihood of respiratory infections, evidenced by an odds ratio of 0.523 (*p* < 0.001), indicating that preschool-age children receiving a dose of vitamin D higher than 600 IU/week were about half as likely to experience respiratory infections compared to those who did not. Furthermore, no significant associations were found between sun exposure, daily sunscreen use, and the incidence of respiratory infections. Conclusively, this study underscores the potential role of vitamin D in helping the immune system against respiratory infections in preschool-age children. The observed protective effect of vitamin D supplementation suggests a potential public health strategy to mitigate the incidence of respiratory infections in preschool children on top of the already known benefits.

## 1. Introduction

Vitamin D is synthesized in the skin from 7-dehydrocholesterol when exposed to ultraviolet B (UVB) radiation from sunlight, converting it to cholecalciferol (vitamin D3). Additionally, it can be absorbed through the diet from foods such as fatty fish, egg yolks, and fortified products [1]. Once vitamin D3 is produced or ingested, it undergoes two hydroxylation steps to become biologically active. The first occurs in the liver, where vitamin D3 is converted into 25-hydroxyvitamin D (25(OH)D) by the enzyme 25-hydroxylase. This form of vitamin D is the main circulating form and is what is commonly measured in laboratory analyses to assess vitamin D status. The second hydroxylation takes place in the kidneys, involving the enzyme 1α-hydroxylase, which converts 25(OH)D into the physiologically active form, 1,25-dihydroxyvitamin D (1,25(OH)_2_D). This active form is crucial for calcium absorption and bone health. The measurement of 25(OH)D is preferred in clinical settings because it has a longer half-life and reflects overall vitamin D reserves more accurately than the highly regulated 1,25(OH)_2_D [1,2].

Vitamin D, a fat-soluble vitamin, plays an important role in calcium homeostasis and bone metabolism [1,2,3]. Beyond these traditional roles, emerging evidence suggests a significant impact of vitamin D on the immune system, particularly in the prevention of infections [3,4,5]. Epidemiological studies have consistently shown a link between vitamin D deficiency and increased susceptibility to infectious diseases, including respiratory infections [6,7,8]. This association is of particular interest in preschool children who are at a higher risk of developing respiratory infections due to their still-developing immune systems and high exposure rates in communal settings, such as daycare and preschool [9,10,11].

Respiratory infections, ranging from the common cold to more severe conditions like pneumonia, represent a leading cause of morbidity among preschool-age children worldwide [12,13,14]. The burden of these infections is not only reflected in the immediate health of the child but also in increased healthcare costs, parental anxiety, and lost days from school and work. Vitamin D‘s potential to enhance innate and adaptive immune responses suggests a promising adjunctive strategy to reduce the incidence and severity of respiratory infections in this vulnerable population [15,16,17]. In this context, several large studies and meta-analyses observed the safety and overall protective role of vitamin D supplements and higher 25(OH)D levels against respiratory infections [18,19].

Despite the plausibility of vitamin D‘s role in modulating immune function, the clinical efficacy of vitamin D supplementation as a preventive measure against respiratory infections in children remains a subject of debate [20,21]. Studies have yielded mixed results, with some showing a significant reduction in the incidence of infections with vitamin D supplementation, while others report no substantial benefits [22,23]. This inconsistency may be attributed to variations in the study design, population, vitamin D dosing, and baseline vitamin D status of participants.

Real-world data underscore the prevalence of vitamin D deficiency in preschool children, which is attributed to factors, such as insufficient dietary intake, limited sun exposure, and the increased use of sunscreen. In the United States, the National Health and Nutrition Examination Survey (NHANES) has documented that approximately 70% of preschool children do not meet the recommended dietary allowance for vitamin D [24,25]. Similar trends are observed globally, emphasizing the need for effective public health strategies to address this deficiency.

Research suggests that vitamin D can influence the production of antimicrobial peptides. such as cathelicidin, which is part of the innate immune system and can annihilate respiratory pathogens [26,27]. In preschool-age children, who are particularly susceptible to respiratory infections due to their developing immune systems and frequent exposure to pathogens in group settings, maintaining optimal levels of vitamin D can be critical. The frequent occurrence of vitamin D insufficiency in this age group further justifies the need for supplementation as a preventative health measure. Therefore, supplementing with vitamin D may not only correct the high prevalence of insufficiency but also enhance the immune responses that are essential in protecting these children from respiratory infections. Therefore, the current study aims to determine the serum vitamin D levels in preschool children and assess their role and supplementation in preventing respiratory infections in this population. By evaluating the efficacy of vitamin D in this context, this study seeks to provide a comprehensive understanding of its potential as a preventive strategy against respiratory infections in preschool children.

## 2. Materials and Methods

### 2.1. Study Design and Ethical Considerations

This cross-sectional study was conducted over a period of 1.5 years, spanning from May 2022 to December 2023, and taking place at the County Clinical Hospital from Timisoara, Romania, and private clinics, focusing on the evaluation of preschool children with and without respiratory infections based on their vitamin D status.

This study adhered strictly to the ethical standards set by the institutional research committee and was in alignment with the principles of the 1964 Helsinki Declaration and its subsequent amendments concerning ethical standards in medical research. The research protocol received thorough review and approval from the Ethical Committee for Scientific Research at the “Victor Babes” University of Medicine and Pharmacy Timisoara. The approval, granted in 2022, was documented under approval number 65.

Consent was meticulously obtained by fully informing the parents or legal guardians of the study’s scope, potential risks, and benefits. Additionally, age-appropriate explanations were provided to the children to gain their assent. All processes were designed to adhere strictly to ethical standards, ensuring both the protection and comfort of the young participants throughout the study’s duration.

### 2.2. Inclusion and Exclusion Criteria

The inclusion criteria for this study comprised the following: (1) preschool children aged between 2 and 5 years; (2) children whose parents agreed to determine the status of their vitamin D serum levels, allowing for an assessment of their vitamin D status and its potential correlation with respiratory health; (3) the completion of a background check regarding the vitamin D status and influencing factors; (4) children who experienced at least one documented respiratory infection in the past year, providing a direct measure to evaluate the protective role of vitamin D against respiratory infections. Conversely, the exclusion criteria were distinctly defined to maintain the study’s focus and integrity as follows: (1) children with chronic respiratory conditions, such as asthma or cystic fibrosis, due to the potential confounding effect on the study’s outcomes; (2) children diagnosed with any condition affecting vitamin D metabolism (e.g., renal disease, hyperparathyroidism), which could independently influence the study’s findings; (3) cases where medical records were not available or consent for using these records in the study was not obtained; (4) the refusal to measure the vitamin D levels in private clinics at the expense of the participants; (5) and children in the acute phase of respiratory infection.

Children with chronic conditions such as asthma were excluded to avoid complications that could arise from their underlying health issues, which might affect the study’s outcomes in relation to respiratory infections and vitamin D‘s effect on immune function. This exclusion helped when isolating the impact of vitamin D on otherwise healthy children, making the results more interpretable regarding the direct relationship studied. To address potential selection bias, participants were recruited from a wide demographic range using multiple platforms, such as pediatric clinics, community centers, and schools.

### 2.3. Laboratory Analysis

Within the framework of this study, serum levels of 25-hydroxyvitamin D (25(OH)D) were quantified through the immunoassay method using the COBAS INTEGRA 400 PLUS automated analyzer. According to existing recent guidelines [28], reference values for 25(OH)D levels are defined as follows: (1) the optimal level ranges from 30 to 55.5 ng/mL; (2) the insufficient level ranges from 21 to 29 ng/mL; (3) deficiency is indicated by levels of 20 ng/mL or less; and (4) severe deficiency is marked by levels of 16 ng/mL or less. Each participant’s 25-OHD level was measured at physicians’ recommendations. A wide range of variables were analyzed to investigate the relationship between vitamin D supplementation and the incidence of respiratory infections among preschool-age children. The collected data encompassed basic demographic details, physiological measurements, vitamin D metabolism markers, inflammatory and infection markers, complete blood count elements, and health outcomes related to infections and hospitalizations.

Demographic and physiological parameters, such as age, gender, body mass index (BMI), height, and weight, were recorded, along with development status (normal, underweight, wasting, stunting) and background (urban or rural). The study also investigated daily calorie intake and nutritional vitamin D supplementation status to determine dietary impacts. Markers critical for understanding vitamin D metabolism, including 25-OHD, PTH (parathyroid hormone), calcitonin, serum calcium, serum phosphate, and creatinine levels, were measured.

Inflammatory and infection markers, such as CRP (C-reactive protein), ferritin, ESR (erythrocyte sedimentation rate), and iron levels, in addition to complete blood count components including WBC (white blood cells), RBC (red blood cells), lymphocytes, hemoglobin, hematocrit, and platelets, were evaluated. Measurements of acid uric and magnesium were also taken. Information regarding vaccinations, the presence of infection, type of infection, site of infection, and the use of antibiotics were used.

### 2.4. Statistical Analysis

Data management and analysis were conducted utilizing the statistical software SPSS version 26.0 (SPSS Inc., Chicago, IL, USA). Continuous variables were represented as the mean ± standard deviation (SD), while categorical variables were expressed in terms of frequencies and percentages. Student’s *t*-test was used to compare two means between the continuous data. The Chi-square test was utilized for the categorical variables. The best cutoff value, sensitivity, specificity, Area Under Curve (AUC), and the Receiver Operating Characteristic were calculated to determine the prediction value of the proposed parameters. A *p*-value threshold of less than 0.05 was set for statistical significance. All results were double-checked through independent re-analysis to ensure accuracy and reliability.

## 3. Results

### Background Characteristics

The study encompassed a sample of 215 children, split into two groups: those with recurrent respiratory infections (*n* = 141) and those without (*n* = 74). Regarding age, the mean age of children with recurrent infections was slightly higher (3.78 years) compared to those without recurrent infections (3.67 years), although the difference was not statistically significant (*p* = 0.483).

A notable finding emerged in the assessment of vitamin D supplementation, where a significant difference was observed. Only 22.70% of children with recurrent infections received vitamin D supplementation above 600 UI/week compared to 59.46% in the non-recurrent group, yielding a highly significant *p*-value (<0.001). Further analysis of the type of respiratory infection (upper versus lower tract) did not yield significant differences (*p* = 0.503). Sun exposure time and daily sunscreen use did not significantly correlate to respiratory infection recurrence, with *p*-values indicating no significant association, as seen in Table 1.

The concentration of 25-hydroxyvitamin D_3_ (25-OHD) showed a significant difference between the groups. Children with recurrent respiratory infections had a lower mean concentration of 24.5 ng/mL, falling below the optimal range and significantly lower than the 29.7 ng/mL observed in children without recurrent infections (*p* < 0.001). Parathyroid hormone (PTH) levels were also higher in the group with recurrent infections, with a mean value of 55.2 ± 15.3 pg/mL compared to 48.7 ± 14.8 pg/mL in the non-recurrent group, which was statistically significant (*p* = 0.023). Other parameters, including calcitonin, calcium, phosphate, and creatinine levels, were within normal ranges and did not show significant differences between the two groups.

However, C-reactive protein levels were significantly higher in children with recurrent respiratory infections (6.8 mg/L) compared to those without (3.1 mg/L), with a *p*-value of <0.001. ESR showed a moderate but statistically significant difference, with higher rates in the recurrent infection group (18.5 mm/h) compared to the non-recurrent group (14.7 mm/h), indicating a higher level of inflammation (*p* = 0.037). The study also found a significant difference in white blood cell (WBC) counts, with higher counts in children with recurrent respiratory infections (8.3 × 10^9^/L) compared to those without (7.2 × 10^9^/L) (*p* = 0.004). The vitamin D assessment further highlighted the nutritional status, showing a significantly higher percentage of children with optimal vitamin D levels in the non-recurrent group (33.78%) compared to the recurrent group (12.77%) (*p* < 0.001), as presented in Table 2. Conversely, a higher proportion of vitamin D deficiency was noted in children with recurrent infections.

A clear gradient was observed in the levels of 25-hydroxyvitamin D_3_ (25-OHD), with children experiencing infections only once after having the highest mean concentration of 29.7 ± 6.1 ng/mL, which decreased to 25.2 ± 5.4 ng/mL in children with two to three infections, and further to 22.8 ± 4.9 ng/mL in those with more than three infections (*p* < 0.001). C-reactive protein levels escalated markedly with the frequency of infections, from 3.1 ± 1.2 mg/L in the group with one infection to 8.7 ± 3.3 mg/L in the group with more than three infections (*p* < 0.001). ESR followed a similar pattern, providing additional evidence of increased inflammatory activity with more frequent infections (*p* = 0.002). White blood cell counts also showed a significant increase, correlating with the frequency of infections (*p* < 0.001).

Children who experienced respiratory infections only once had the highest proportion of optimal vitamin D levels (33.78%), which is within the range of 30 to 55.5 ng/mL. In the groups with multiple respiratory infections, the proportion of optimal vitamin D levels was significantly diminished to 13.48% and 9.62%, respectively. Conversely, the proportion of children classified as vitamin D deficient (levels of 20 ng/mL or less) escalated with the frequency of respiratory infections. This trend is especially notable in children who had more than three infections, where 38.46% were found to be vitamin D deficient, compared to 31.46% in the group with two to three infections and only 16.22% in the once-infected group. Moreover, the analysis extended to children with severe vitamin D deficiency (levels below 16 ng/mL), although the increases in proportions across groups from those infected once (4.05%) to those with more than three infections (9.62%) did not reach the same level of statistical significance, as presented in Table 3.

**Figure 1:**
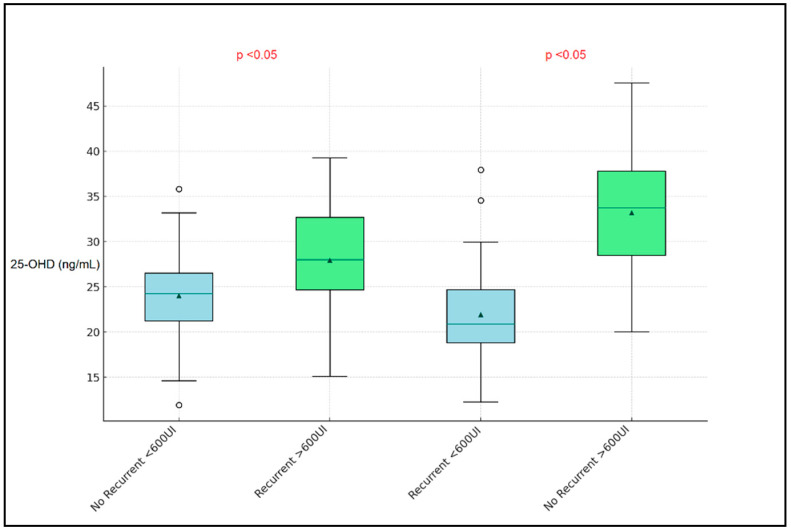
Boxplot analysis of vitamin D levels between preschool children with and without recurrent respiratory infections in the past 12 months (non-recurrent indicates only 1 respiratory infection).

Figure 1 presents the recurrent versus non-recurrent respiratory infections and compares these based on vitamin D supplementation levels (<600 UI weekly versus >600 UI weekly), illustrating a notable distinction in median vitamin D levels between the groups. Specifically, children receiving more than 600 UI of vitamin D weekly tended to have higher median vitamin D levels regardless of their infection recurrence status. The groups receiving less than 600 UI and more than 600 UI of vitamin D weekly displayed median vitamin D levels of 24.5 ng/mL and 29.7 ng/mL, respectively.

**Figure 2:**
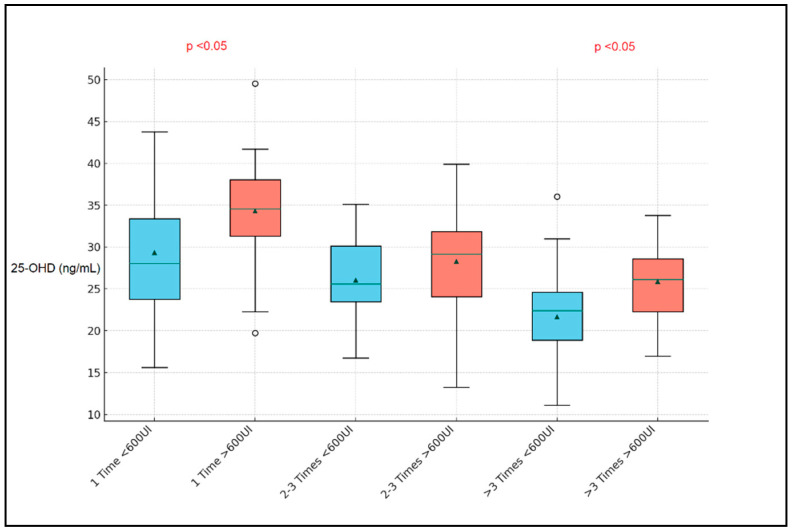
Boxplot analysis by number of respiratory infections in the past 12 months.

It was observed that groups with more frequent respiratory infections exhibited lower median vitamin D levels, accentuating the potential protective role of vitamin D against such infections. Notably, the subgroup experiencing more than three respiratory infections and receiving less than 600 UI of vitamin D, weekly, showcased a median vitamin D level of 22.8 ng/mL with an IQR of 4.9 ng/mL, compared to their counterparts receiving more than 600 UI, who exhibited a slightly higher median of 26.15 ng/mL and an IQR of 6.31 ng/mL (Figure 2).

Children with severe vitamin D deficiency levels were significantly more likely to suffer from respiratory infections, with an odds ratio (Exp(B)) of 2.967, indicating that they were almost three times as likely to have infections compared to those with optimal vitamin D levels (*p*-value < 0.001).

Vitamin D supplementation greater than 600 IU/week was associated with a significant decrease in the likelihood of respiratory infections, with an odds ratio of 0.523 (*p*-value < 0.001), indicating that children receiving this level of supplementation were about half as likely to experience respiratory infections as those who did not receive supplementation, as presented in Table 4.

## 4. Discussion

### 4.1. Literature Findings

The findings from our cross-sectional study found a potentially protective role of vitamin D supplementation in reducing the incidence of respiratory infections among preschool-age children. An interesting aspect of this study was the demonstrable effect of vitamin D supplementation above 600 IU/week, which significantly reduced the likelihood of respiratory infections. This correlation supports the hypothesis that vitamin D can have an important role in enhancing immune resilience in this age group.

Critical to our discussion is the contrast in outcomes based on vitamin D status. Children with severe vitamin D deficiency were found to be almost three times more likely to experience respiratory infections compared to those with optimal levels. This significant finding not only highlights the importance of maintaining adequate vitamin D levels for immune health but also indicates the need for targeted interventions in populations at risk of deficiency. Interestingly, our study also found a non-significant relationship between sun exposure, daily sunscreen use, and the incidence of respiratory infections. This finding suggests that the protective effects of vitamin D on respiratory health are not undermined by limited sun exposure or sunscreen use, which are critical factors for skin cancer prevention. It reinforces the importance of dietary supplementation as a reliable method for maintaining adequate vitamin D levels, particularly in regions with limited sunlight or in populations where outdoor activities are minimized.

Moreover, the study’s outcomes emphasize the importance of vitamin D supplementation as a public health strategy. The substantial reduction in infection with supplementation greater than 600 IU/week underscores the potential for vitamin D as a preventive measure against respiratory infections. These findings advocate for the incorporation of vitamin D supplementation guidelines into public health policies, especially for children at preschool age, to enhance their overall health status and immune resilience. Further research is warranted to explore the optimal levels of supplementation and to evaluate the long-term benefits of maintaining adequate vitamin D levels in preventing respiratory and other infections in this demographic.

However, in addressing how our findings compare with the existing literature, particularly where they diverge from expected outcomes, it is crucial to consider potential discrepancies. Differences in study design, participant demographics, or methodologies may contribute to these variations. This analysis not only reflects a thorough engagement with the field but also identifies specific areas that warrant further investigation.

The findings from Balan et al. [29] and Esposito et al. [20] converge on the important role of vitamin D in protecting against respiratory tract infections in children, which are a leading cause of morbidity and mortality globally. Balan et al. emphasized the alarming prevalence of vitamin D deficiency among infants and its association with heightened risks of severe respiratory infections, including pneumonia, which is responsible for 19% of all deaths in children under five years old. They advocate for the potential of vitamin D supplementation and improved sun exposure guidelines to significantly reduce these risks; however, no actual threshold values for (25(OH)D) were suggested, reinforcing the novelty that our study brings. On the other hand, Esposito et al. [20]’s review further substantiates the immunomodulatory properties of vitamin D and its link to various RTIs in children, noting the absence of a consensus on the definition of vitamin D deficiency and the adequate levels necessary for immune function. They suggest that maintaining serum 25-hydroxycholecalciferol levels between 20 ng/mL and 50 ng/mL could provide protective immunomodulatory effects against RTIs, including tuberculosis and severe bronchiolitis, similar to our findings where serum levels above a threshold of 26.15 ng/mL were considered protective for RTIs, and with the additional dose-dependent impact of more than 600 UI/week.

Contrary to our findings, Şişmanlar et al.’s case–control study [30] found no significant correlation between vitamin D levels and the incidence or severity of lower respiratory tract infections despite a widespread prevalence of vitamin D deficiency/insufficiency among the children studied. On the contrary, Nicolae et al. [31], through a literature review, highlighted the potential immunomodulatory and antiviral effects of vitamin D, suggesting that its supplementation might lower the risk of acute respiratory tract infections, including COVID-19. While Şişmanlar et al. caution against assuming the direct protective role of vitamin D against such infections due to their findings, Nicolae et al. advocate for the potential benefits of vitamin D in mitigating the severity of RTIs, urging further research to solidify these preliminary observations.

Similarly to our findings, Xiao et al.’s study on children with recurrent respiratory tract infections showed a notable improvement in treatment outcomes with vitamin D supplementation, including a significant total effective treatment rate of 96.67% in the vitamin D group versus 71.19% in the control group [32]. This group also saw improvements in immune function indicators, such as IgA, IgG, IgM, and CD4+ levels, and a reduction in the number of respiratory infection episodes. Abioye et al. [33], examining adults, found that vitamin D supplementation slightly reduced the risk of acute respiratory tract infections with a risk ratio of 0.97 and shortened symptom duration of 6%. Their review also highlights the effectiveness of vitamin C in reducing the risks of RTIs (RR = 0.96) and zinc, significantly shortening symptom duration by 47% and underscoring the potential of micronutrients to enhance immune responses against respiratory infections across different age groups.

Jolliffe et al. [18] and Martineau et al. [34] both underscore the efficacy of vitamin D supplementation in reducing the risk of acute respiratory infections through their meta-analyses, albeit with nuanced differences in their findings. Jolliffe et al., analyzing data from 45 RCTs and involving over 73,000 participants, concluded that vitamin D supplementation offers a modest protective effect against acute respiratory infections (OR 0.91, 95% CI 0.84 to 0.99), with daily doses of 400–1000 IU for up to 12 months being particularly effective. Martineau et al. [34], on the other hand, focusing on 25 RCTs with around 11,000 participants, highlighted that vitamin D supplementation significantly reduced the risk of ARIs (adjusted odds ratio 0.88, 95% CI 0.81 to 0.96), especially in those very deficient in vitamin D (baseline 25[OH]D levels < 25 nmol/L) and in recipients of daily or weekly doses without bolus doses. While both analyses agree on the protective role of vitamin D against ARIs and its safety, Martineau et al. provide a more detailed insight into the effectiveness based on baseline vitamin D levels and dosing strategies.

Other studies, such as those conducted by Jat et al. [35] and Buendia et al. [36], provide complementary insights into the role of vitamin D in pediatric respiratory health, underlining both clinical and economic benefits. Jat et.al’s systematic review points to a significant correlation between low vitamin D levels and the increased incidence and severity of lower respiratory tract infections in children, suggesting a potential protective role of vitamin D against such infections. Buendia et al., through a cost-utility analysis, further bolster the argument for vitamin D supplementation by demonstrating its cost-effectiveness in preventing acute respiratory infections in children, showcasing not only a reduction in healthcare costs (USD 1354 with supplementation vs. USD 1948 without) but also a slight improvement in quality-adjusted life years (QALYs) (0.99 with vitamin D supplementation vs. 0.98 without). Regarding the implications for a public health policy, although our study did not include a detailed analysis of the costs of healthcare burden, it should be considered in the context of the universal free healthcare in Romania, where this study took place. Nevertheless, a campaign to supplement preschool-age children’s diet with at least 600 UI of vitamin D can be considered according to our findings.

Our study, along with corroborating research, emphasizes vitamin D‘s significant role in combating respiratory infections in children. We found that children with recurrent infections often had lower vitamin D levels, highlighting the potential protective effect of vitamin D supplementation, particularly at doses over 600 IU/week. This aligns with broader research suggesting vitamin D‘s efficacy in preventing respiratory ailments across different demographics. Our findings advocate for vitamin D supplementation as a viable public health strategy to enhance immune defense among children. Given the accessible nature and potential benefits of such supplementation, further research should aim to optimize vitamin D intake guidelines for pediatric populations.

The current study findings might not be readily applicable to the general population due to the observed lack of the effect of sun exposure, considering the temperate climate of this country of study and the relatively minor differences in 25-OHD levels between groups, which suggest that precise measurements are essential to discern any significant differences. Moreover, the perceived limited availability of the study suggests that supplementing with vitamin D could be a more feasible solution. To validate these findings in diverse regions, a comparison between groups with and without supplements may offer clearer insights.

### 4.2. Limitations and Future Perspectives

Despite these insightful findings, our study acknowledges several limitations that merit consideration. First, the cross-sectional design limits the ability to infer causality between vitamin D status or supplementation and the incidence of respiratory infections. Longitudinal studies are necessary to establish a temporal relationship and causality. Additionally, the reliance on parental reports for data on sun exposure and supplementation intake could introduce recall bias, potentially affecting the accuracy of these variables. Another limitation is the study’s geographic focus on preschool-age children from specific locations within Romania, which may limit the generalizability of the findings to other climates or populations with different dietary habits and lifestyle factors. Furthermore, this study did not account for all potential confounding variables, such as seasonal variations in vitamin D, indoor air quality, exposure to secondhand smoke, and socioeconomic status, which could influence the risk of respiratory infections.

Based on the findings of this study, future research should explore the optimal dosage and duration of vitamin D supplementation necessary to prevent respiratory infections in preschool-age children effectively. Additionally, longitudinal studies could investigate the long-term effects of maintaining higher 25(OH)D serum levels through regular supplementation, especially in different climatic and geographical settings. Given the observed significant impact of vitamin D on reducing respiratory infections, further studies should also consider the interaction between vitamin D supplementation and other preventive health measures, such as vaccination and hygiene practices, to develop comprehensive public health strategies.

## 5. Conclusions

The findings from this research highlight the potential benefits of vitamin D in boosting the immune defense against respiratory infections among preschool-age children. Evidenced by the positive impact of vitamin D supplementation, this approach appears to be a promising public health intervention aimed at reducing the frequency of such infections within susceptible pediatric groups. This study supports the idea of integrating vitamin D supplementation recommendations into broader public health policies, especially targeting children in areas with inadequate exposure to sunlight. To fully realize the health benefits for this sensitive population group, additional studies are necessary to refine vitamin D supplementation practices, ensuring they are tailored to effectively enhance the pediatric immune system.

## Figures and Tables

**Table 1:** Comparison of background characteristics.

Variables	Recurrent Respiratory Infections (*n* = 141)	No Recurrent Respiratory Infections (*n* = 74)	*p*-Value
Age (mean ± SD)	3.78 ± 0.95	3.67 ± 1.03	0.483
Age category, *n* (%)			0.675
2–3 years	68 (48.23%)	32 (43.24%)	
4–5 years	73 (51.77%)	42 (56.76%)	
BMI (mean ± SD)	16.3 ± 1.6	16.5 ± 1.3	0.389
Development, *n* (%)			
Normal	108 (76.60%)	56 (75.68%)	0.847
Underweight	16 (11.35%)	12 (16.22%)	0.423
Overweight	11 (7.80%)	5 (6.76%)	0.765
Low weight for height	4 (2.84%)	1 (1.35%)	0.621
Low height for age	2 (1.42%)	0 (0.00%)	0.486
Place of living			0.822
Rural	58 (41.13%)	29 (39.19%)	
Urban	83 (58.87%)	45 (60.81%)	
Daily calorie intake, *n* (%)			
Low (< 1400 kcal)	29 (20.57%)	16 (21.62%)	0.922
Moderate (1400–1600 kcal)	68 (48.23%)	36 (48.65%)	0.966
High (>1600 kcal)	44 (31.21%)	22 (29.73%)	0.882
Vitamin D supplementation (>600 UI/week), *n* (%)			4.8 × 10^−5^
Yes	32 (22.70%)	44 (59.46%)	
No	109 (77.30%)	30 (40.54%)	
Number of respiratory infections in the past 12 months, *n* (%)			3.2 × 10^−5^
1	0 (0.00%)	74 (100.00%)	
2–3	89 (63.12%)	0 (0.00%)	
>3	52 (36.88%)	0 (0.00%)	
Type of respiratory infection			0.503
Upper tract infection	117 (83.00%)	64 (86.49%)	
Lower tract infection	24 (17.00%)	10 (13.51%)	
Sun exposure per day, *n* (%)			
5–15 min	52 (36.88%)	25 (33.78%)	0.744
15–30 min	59 (41.84%)	34 (45.95%)	0.679
>30 min	30 (21.28%)	15 (20.27%)	0.888
Daily sunscreen use, *n* (%)			
Frequently	23 (16.31%)	10 (13.51%)	0.648
Often	44 (31.21%)	28 (37.84%)	0.556
Rarely	58 (41.13%)	26 (35.14%)	0.419
Never	16 (11.35%)	10 (13.51%)	0.764
Antibiotic use in the past 6 months, *n* (%)			0.257
Yes	81 (57.45%)	37 (50.00%)	
No	60 (42.55%)	37 (50.00%)	

SD—standard deviation; BMI—body mass index; adjusted significance level (*p*-value threshold) after Bonferroni correction = 0.0041; non-recurrent indicates only 1 respiratory infection.

**Table 2:** Comparison of laboratory parameters between preschool children with and without recurrent respiratory infections.

Variables (Mean ± SD)	Normal Range	Recurrent Respiratory Infections (*n* = 141)	No Recurrent Respiratory Infections (*n* = 74)	*p*-Value
25-OHD (ng/mL)	30–55.5	24.5 ± 5.3	29.7 ± 6.1	3.9 × 10^−4^
PTH (pg/mL)	15–65	55.2 ± 15.3	48.7 ± 14.8	0.023
Calcitonin (pg/mL)	<10	7.3 ± 2.5	6.9 ± 2.2	0.311
Calcium (mg/dL)	8.8–10.8	9.4 ± 0.8	9.5 ± 0.7	0.542
Phosphate (mg/dL)	3.4–5.5	4.2 ± 0.5	4.3 ± 0.4	0.376
Creatinine (mg/dL)	0.3–0.7	0.5 ± 0.1	0.5 ± 0.1	0.890
CRP (mg/L)	<5	6.8 ± 2.3	3.1 ± 1.2	6.1 × 10^−6^
Ferritin (ng/mL)	15–150	60.3 ± 25.4	55.1 ± 22.3	0.148
ESR (mm/h)	0–20	18.5 ± 8.3	14.7 ± 7.9	0.037
Iron (µg/dL)	50–120	85.2 ± 23.7	91.3 ± 25.1	0.153
WBC (×10^9^/L)	4.0–10.0	8.3 ± 2.1	7.2 ± 1.8	0.004
RBC (×10^12^/L)	4.5–5.5	4.8 ± 0.6	4.9 ± 0.5	0.272
Lymphocytes (%)	20–40	35.2 ± 8.4	37.1 ± 7.9	0.154
Neutrophils (%)	40–60	54.6 ± 7.5	52.8 ± 6.9	0.088
Hemoglobin (g/dL)	11.0–14.0	12.3 ± 1.2	12.5 ± 1.1	0.229
Platelets (×10^9^/L)	150–450	310.5 ± 80.2	325.3 ± 75.4	0.197
Vitamin D assessment, *n* (%)				
Optimal	30–55.5 ng/mL	18 (12.77%)	25 (33.78%)	5.0 × 10^−4^
Insufficient	21–29 ng/mL	63 (44.68%)	34 (45.95%)	0.881
Deficient	20–16 ng/mL	52 (36.88%)	12 (16.22%)	6.6 × 10^−5^
Severe deficiency	<16 ng/mL	8 (5.67%)	3 (4.05%)	0.717

SD—Standard deviation; optimal level ranges from 30 to 55.5 ng/mL; insufficient level ranges from 21 to 29 ng/mL; deficiency is indicated by levels of 20 ng/mL or less; severe deficiency is marked by levels of 16 ng/mL or less. 25-OHD—25-hydroxyvitamin D_3_; PTH—parathyroid hormone; CRP—C-reactive protein; ESR—erythrocyte sedimentation rate; WBC—white blood cells; RBC—red blood cells; adjusted significance level (*p*-value threshold) after Bonferroni correction = 0.0025; non-recurrent indicates only 1 respiratory infection.

**Table 3:** Vitamin D and laboratory assessment based on the prevalence of respiratory infections in preschool children in the past 12 months.

Variables (Mean ± SD)	1 Time (*n* = 74)	2–3 Times (*n* = 89)	>3 Times (*n* = 52)	*p*-Value
25-OHD (ng/mL)	29.7 ± 6.1	25.2 ± 5.4	22.8 ± 4.9	4.8 × 10^−4^
PTH (pg/mL)	48.7 ± 14.8	54.9 ± 15.1	58.3 ± 16.2	0.004
Calcitonin (pg/mL)	6.9 ± 2.2	7.1 ± 2.3	7.5 ± 2.4	0.218
Calcium (mg/dL)	9.5 ± 0.7	9.3 ± 0.8	9.2 ± 0.9	0.076
Phosphate (mg/dL)	4.3 ± 0.4	4.1 ± 0.5	4.0 ± 0.6	0.037
Creatinine (mg/dL)	0.5 ± 0.1	0.5 ± 0.1	0.6 ± 0.1	0.019
CRP (mg/L)	3.1 ± 1.2	6.5 ± 2.1	8.7 ± 3.3	4.2 × 10^−5^
Ferritin (ng/mL)	55.1 ± 22.3	61.7 ± 25.1	67.9 ± 27.5	0.031
ESR (mm/hr)	14.7 ± 7.9	18.4 ± 8.2	21.6 ± 9.4	0.002
Iron (µg/dL)	91.3 ± 25.1	86.4 ± 24.8	82.1 ± 23.6	0.053
WBC (×10^9^/L)	7.2 ± 1.8	8.4 ± 2.0	9.1 ± 2.3	3.9 × 10^−5^
RBC (×10^12^/L)	4.9 ± 0.5	4.8 ± 0.6	4.7 ± 0.7	0.127
Lymphocytes (%)	37.1 ± 7.9	34.5 ± 8.2	32.3 ± 8.5	0.010
Neutrophils (%)	52.8 ± 6.9	55.4 ± 7.2	58.2 ± 7.5	0.003
Hemoglobin (g/dL)	12.5 ± 1.1	12.2 ± 1.2	11.9 ± 1.3	0.017
Platelets (×10^9^/L)	325.3 ± 75.4	308.2 ± 80.1	296.7 ± 82.3	0.044
Vitamin D assessment, *n* (%)				
Optimal	25 (33.78%)	12 (13.48%)	5 (9.62%)	3.6 × 10^−4^
Insufficient	34 (45.95%)	41 (46.07%)	22 (42.31%)	0.912
Deficient	12 (16.22%)	28 (31.46%)	20 (38.46%)	0.001
Severe deficiency	3 (4.05%)	8 (8.99%)	5 (9.62%)	0.258

SD—Standard deviation; optimal level ranges from 30 to 55.5 ng/mL; insufficient level ranges from 21 to 29 ng/mL; deficiency is indicated by levels of 20 ng/mL or less; severe deficiency is marked by levels of 16 ng/mL or less. 25-OHD—25-hydroxyvitamin D_3_; PTH—parathyroid hormone; CRP—C-reactive protein; ESR—erythrocyte sedimentation rate; WBC—white blood cells; RBC—red blood cells; adjusted significance level (*p*-value threshold) after Bonferroni correction = 0.0025; and non-recurrent indicates only 1 respiratory infection.

**Table 4:** Regression table.

Predictor	B (Coefficients)	Standard Error	Wald	df	*p*-Value	Exp(B) (Odds Ratio)
Vitamin D Status (Reference: Optimal)						
Insufficient	0.143	0.193	0.549	1	0.469	1.154
Deficient	0.289	0.218	1.756	1	0.185	1.335
Severe Deficiency	1.087	0.304	12.785	1	<0.001	2.967
Vitamin D Supplementation (>600 IU/week)	−0.648	0.176	13.519	1	<0.001	0.523
Sun Exposure (Reference: >30 min)						
5–15 min	0.132	0.251	0.276	1	0.599	1.141
15–30 min	0.098	0.205	0.228	1	0.633	1.103
Daily Sunscreen Use (Reference: Never)						
Frequently	0.061	0.265	0.053	1	0.817	1.063
Often	0.034	0.184	0.034	1	0.853	1.035
Rarely	−0.017	0.147	0.013	1	0.909	0.983

df—degrees of freedom.

## Data Availability

The original contributions presented in the study are included in the article, further inquiries can be directed to the corresponding author.
